# Negative correlation between school bullying and multi-dimensional health in adolescent female migrants: a digital visual analog scale study

**DOI:** 10.3389/fpsyg.2025.1551243

**Published:** 2025-08-14

**Authors:** Yueping Song, Yifei Li, Kai Zhou, Jia Tang, Xuemeng Chen, Gaowang Liu

**Affiliations:** 1 Research Center for Social and Population Development, Renmin University of China, Beijing, China; 2 School of Population and Health, Renmin University of China, Beijing, China; 3 Big Data and Responsible Artificial Intelligence for National Governance, Renmin University of China, Beijing, China; 4 School of International Trade and Economics, Central University of Finance and Economics, Beijing, China; 5 Policy Research Center for Environment and Economy, Ministry of Ecology and Environment of the People's Republic of China, Beijing, China; 6 Department of Anesthesiology, Deyang People's Hospital, Deyang, Sichuan, China; 7 Department of Anesthesiology, Nanfang Hospital, Southern Medical University, Guangzhou, Guangdong, China; 8 Key Laboratory of Precision Anesthesia & Perioperative Organ Protection, Guangzhou, Guangdong, China

**Keywords:** school bullying, female high school students, physiological indicators, psychological distress, digital visual analog scale (VAS), China

## Abstract

**Introduction:**

School bullying poses significant risks to the physiological and psychological health of female adolescent migrants, a vulnerable group often overlooked during their critical developmental period. This study investigates the specific correlations between different types of bullying exposure and multi-dimensional health outcomes among female high school students aged 15 to 18 in China.

**Methods:**

Data were derived from a longitudinal observational cohort, including clinical records and structured survey assessments. We introduced a novel digital visual analog scale (VAS) to quantify psychological tendencies (e.g., depressive symptoms, sleep disturbances) and physiological indicators (e.g., menstrual irregularities, EEG/ECG variations). Participants were categorized into five groups: violent bullying, multiple bullying, verbal bullying, social bullying, and a non-bullied control group. Statistical analyses were performed to compare health outcomes across these groups.

**Results:**

Students subjected to violent and multiple bullying exhibited markedly higher levels of physiological dysfunction and psychological distress compared to the control group. Verbal and social bullying also resulted in significant psychological symptoms. The severity of bullying showed a positive correlation with elevated VAS scores, indicating a dose-response relationship. These findings underscore the profound impact of school bullying on the health of female adolescent migrants.

**Discussion:**

The results highlight an urgent need for targeted early detection and intervention strategies for this population. The digital VAS developed in this study proves to be an effective and sensitive tool for educators and clinicians to monitor and address the adverse health outcomes of bullying. These findings call for integrated support systems that address both the physical and mental health of vulnerable adolescents.

## 1 Introduction

Urbanization is a defining global trend of the 21st century, with rapid urban growth seen in developing countries (Lei et al., 2022). China exemplifies this phenomenon, experiencing unprecedented internal migration and urban expansion. By 2020, ~493 million people, which are around 35% of China's population, lived away from their registered households. This includes 376 million people who migrated temporarily or permanently without changing their official household registration, commonly referred to as “floating population,” marking a nearly 70% increase in the last decade (MacLachlan and Gong, 2022). This demographic shift has profound social, economic, and health implications.

China's unique household registration system (hukou) has historically constrained migrants' access to social services and welfare, although reforms have been gradually introduced to ease these restrictions (Chan and Buckingham, 2008). Nevertheless, the floating population, especially youth, faces significant challenges related to social integration, resource accessibility, and identity formation (Lu and Wang, 2013; Wu and Zheng, 2018; Tang et al., 2022). Female adolescents migrating from rural to urban areas during their critical developmental years constitute a vulnerable subgroup often overlooked in public health research (Bahar et al., 2024; Nicolle, 2022). They encounter complex environmental, social, and psychological stressors that can adversely impact their health and wellbeing (Cheung, 2014; Fang et al., 2016; Chen and Qu, 2021; Xie and Cui, 2022).

Among these stressors, school bullying has emerged as a pervasive issue with documented detrimental effects on youths' mental and physical health globally (Arslan et al., 2021; Hysing et al., 2021; Källmén and Hallgren, 2021; Zhao et al., 2024; Feng et al., 2024). Bullying, including physical, verbal, relational, and cyber forms, has been linked to long-term negative outcomes such as depression, anxiety, somatic complaints, and impaired academic performance (Malhi and Bharti, 2021; Menken et al., 2022; Zhao et al., 2024; Anan et al., 2025). From a socioecological perspective, bullying is influenced by individual, family, school, and community factors, emphasizing the need for multifaceted intervention strategies (Merrin et al., 2018). However, much of the extant literature on bullying focuses on general adolescent populations, with limited attention to migrant adolescents, especially female individuals.

Furthermore, Bronfenbrenner's bioecological model underscores the critical role of proximal environments, such as schools, in shaping adolescent development, particularly during sensitive growth periods (Bronfenbrenner, 2005; Tudge et al., 2009). In the case of migrant adolescent girls, school environments could present unique risks due to social exclusion, cultural dissonance, and discrimination, exacerbating their vulnerabilities to bullying and its consequences (Ames and Yon, 2022; Bahar et al., 2024; Nicolle, 2022). Despite this theoretical foundation, there is a notable paucity of empirical research investigating the intersection of migration status, bullying exposure, and multidimensional health outcomes in female adolescents. Existing studies often fail to differentiate based on gender, developmental stage, or migration background, thereby limiting the development of targeted policies and interventions.

To fill this significant gap, our study leveraged hospital-derived data and standardized bullying classifications to conduct an in-depth analysis of the physiological and psychological impacts of bullying among migrant female high school students. We employed a novel digital visual analog scale (VAS) that enabled quantification of both psychological tendencies, such as depressive symptoms, sleep disturbances, and learning inefficiency, and physiological indicators, such as menstrual irregularities and electrophysiological variations [electroencephalogram (EEG) and electrocardiogram (ECG)]. This study applied robust statistical modeling to elucidate associations across bullying types and health domains.

This study addresses an urgent need for context-specific, gender-sensitive research to develop early detection, prevention, and intervention programs tailored to migrant female adolescents. By advancing our understanding of how school bullying compounds the health challenges faced by this marginalized population, this study offers vital evidence for educators, clinicians, and policymakers aiming to enhance health equity and developmental outcomes during a pivotal life stage.

## 2 Materials and methods

### 2.1 Study design, randomization, and sample description

This study utilized a longitudinal observational cohort design to investigate the associations between school bullying and multidimensional health outcomes in female adolescents experiencing population mobility. The longitudinal framework facilitated the temporal assessment of bullying effects while controlling for confounding variables, thereby providing robust insights into the physiological and psychological impacts of bullying.

Data were derived from a comprehensive baseline survey conducted in 2014 and a follow-up assessment conducted in 2017. Follow-up data were successfully collected from 4,221 students across multiple schools. Additionally, hospital data used in this study were obtained from a medical institution located in eastern China, providing clinical records pertinent to the participants' physiological health indicators. For the current analysis, the focus was restricted to female high school students classified as migrants, especially adolescents who relocated from rural to urban areas and were enrolled in either general senior high schools or secondary technical/vocational institutions. To ensure homogeneity and relevance, participants still in junior high school, those who had discontinued their studies, or those with missing critical variables were excluded. The resulting analytic sample comprised 470 female adolescents who met the inclusion criteria.

Randomization procedures were implemented at the individual student level within sampled classes to minimize selection bias and facilitate comparability across study groups. Specifically, eligible students were randomly selected to participate in the study assessments, ensuring an unbiased representation of bullying exposure within the target population. Group classification was operationalized based on self-reported bullying experiences within the past year, which were obtained via structured questionnaires and confidential interviews. Participants, who reported exposure to at least one form of bullying, such as physical, verbal, social, or cyberbullying, were assigned to the bullying-exposed group, while those who reported no such experiences constituted the control group. This classification strategy allowed for a naturalistic yet rigorous comparison of health outcomes linked to bullying status.

### 2.2 Ethical consideration

This study was reviewed and approved by the Ethics Committee of a tertiary hospital located in eastern China. The approval process adhered to the ethical guidelines set forth by several authoritative bodies, including the National Health and Family Planning Commission's “Measures for the Ethical Review of Biomedical Research Involving Humans (2016)”, the National Medical Products Administration and National Health Commission's “Good Clinical Practice for Drug Trials (2020)”, the “Good Clinical Practice for Medical Device Trials (2016),” the World Medical Association's “Declaration of Helsinki (2013)”, and the Council for International Organizations of Medical Sciences' (CIOMS) “International Ethical Guidelines for Biomedical Research Involving Human Subjects”. Following a thorough review, the Ethics Committee confirmed that this project on the psychological impact of school bullying adheres to the fundamental principles of medical ethics. Informed consent was obtained from all participants, and approval for their participation in this longitudinal project was granted.

### 2.3 Operational definition of bullying and grouping criteria

Bullying, a complex and multifaceted social phenomenon, was operationalized in this study following internationally recognized frameworks adapted to the Chinese context (Huang et al., 2013; Chan and Wong, 2015; Xing et al., 2023). Consistent with prior research emphasizing cross-cultural validity of bullying constructs, bullying behaviors were classified into four distinct types: physical, verbal, social (including relational), and cyberbullying. Data on bullying experiences were obtained through proxy reporting by closest confidants (e.g., parents or siblings) to protect participant confidentiality and minimize reporting bias, as recommended in epidemiological studies of sensitive psychosocial phenomena (Rivers, 2001; Vessey et al., 2014; Fardell et al., 2024).

Physical bullying encompassed overt aggressive acts such as pushing, hitting, or stealing personal belongings. Verbal bullying included insults, public ridicule, and threatening language, while social bullying involved exclusionary behaviors and the spreading of rumors, and cyberbullying consisted of digital harassment, insults, and unauthorized dissemination of private information (Wang et al., 2009, 2012; Johansson and Englund, 2021). Participants who reported any incidence of bullying within the previous year were designated as victims of the corresponding bullying type. Those who experienced multiple concurrent bullying types were classified accordingly to examine compounded effects.

To maintain internal validity and address confounders, the exclusion criteria encompassed female students with congenital or hereditary disorders, prior traumatic injuries unrelated to bullying, and those with incomplete data. This rigorous classification and screening ensured a homogeneous sample for reliable subgroup analyses.

### 2.4 Grouping and experimental classification

Based on existing studies (Wang et al., 2012; Wu and Zheng, 2018), participants were categorized into bullying exposure groups aligned with the logistic growth model framework to capture the heterogeneity of bullying impact on developmental trajectories. The groups were categorized as follows:

- Group A: Experiencing violent (physical) bullying.
- Group B: Experiencing multiple bullying types including physical violence.
- Group C: Experiencing verbal bullying exclusively.
- Group D: Experiencing social and cyberbullying exclusively.
- Group E: Non-bullied control group.

This classification reflects differential psychosocial risk profiles documented in migrant adolescent cohorts, where the severity and multiplicity of bullying exposures correlate with distinct mental and physical health outcomes. The approach allows for a nuanced investigation into bullying typologies beyond binary victimization status.

### 2.5 Data collection procedures and observed indicators

Consistent with the emerging trend in psychosocial epidemiology to leverage quantifiable measures of subjective experiences, this study employed a novel digital visual analog scale (VAS) to assess psychological tendencies and physiological health indicators (Kersten et al., 2012; Price et al., 2012). The VAS was designed as a continuous 20 cm scale, anchored by empirically validated descriptors spanning mild to severe depressive symptomatology, sleep disturbances, and learning inefficiency.

**Figure 1:**
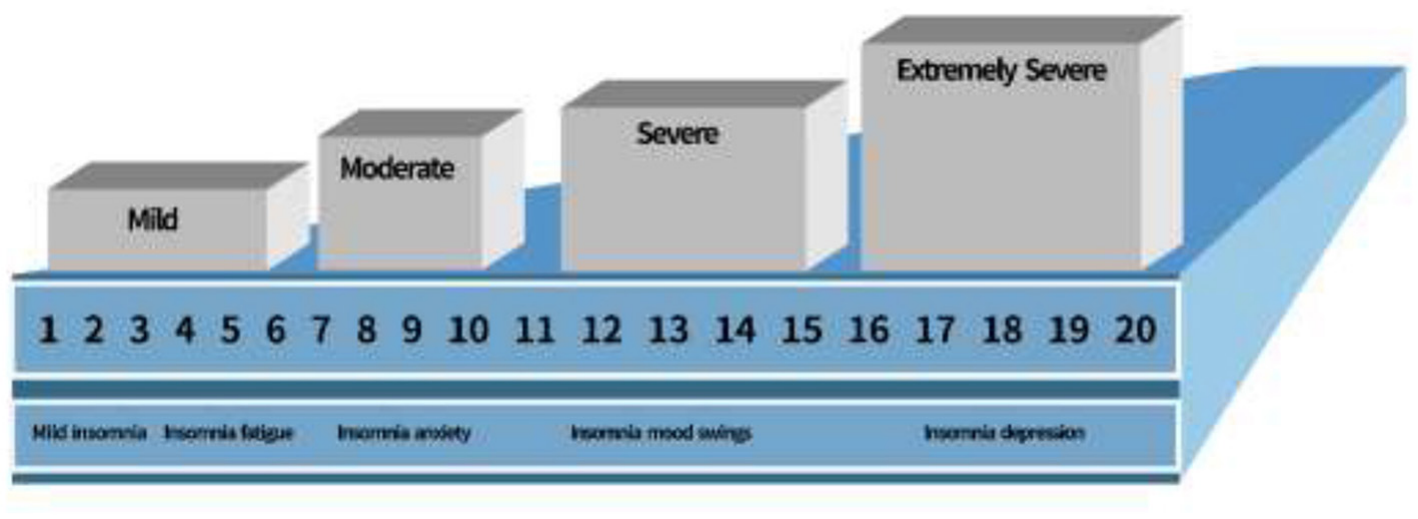
Bar chart depicting levels of insomnia severity from mild to extremely severe, with a scale from 1 to 20. Descriptions under each level include symptoms like fatigue, anxiety, mood swings, and depression. Visual analog digital score of psychological impact on sleep quality. The visual analog digital score of visual analog digital score, an indicator of psychological impact on sleep quality.

Psychological tendencies were operationalized through a validated 13-item instrument assessing affective states over the past 7 days post-bullying exposure, capturing symptoms such as hopelessness, concentration difficulties, mood instability, and social withdrawal. Positive affect items were reverse-coded to enhance sensitivity to psychological distress. Physiological indices focused on menstrual health parameters, including cycle irregularities (delay, hypomenorrhea, or hypermenorrhea), dysmenorrhea, and amenorrhea, complemented by electrophysiological measures such as EEG and ECG variations indicative of autonomic and central nervous system dysregulation. Academic performance was quantitatively measured via the VAS scale, reflecting engagement and stability, ranging from consistent interest and on-time homework completion to marked disengagement and performance decline (Figure 1).

Data collection adhered to standardized protocols with trained interviewers ensuring reliability and minimizing information bias.

### 2.6 Statistical analysis

Data analysis incorporated robust, non-parametric, and parametric methods appropriate for the ordinal and continuous outcome variables. Descriptive statistics summarized demographic and bullying exposure characteristics.

Associations between bullying exposure and health outcomes were evaluated using ordinal logistic regression models, accommodating the ordered nature of psychological and physiological symptom scales. Continuous VAS scores were analyzed via ordinary least squares (OLS) regression, adjusting for potential confounders such as age, school type, and migration status. Model diagnostics confirmed assumptions of proportional odds and homoscedasticity.

All analyses were conducted using Stata 18.0 with a significance threshold set at *p* < 0.05. This analytic framework ensured rigorous and interpretable inference on multifaceted impacts of bullying on migrant adolescent female population.

## 3 Results

A comparison of age, height, and weight among groups A, B, C, D, and E showed no significant differences (*p* > 0.05) (Table 1). This indicates that the demographic variables (age, height, and weight) were comparable across all groups, allowing for a more focused analysis of other factors.

**Table 1:** Sample general data (x ± s).

**Group**	**Age range**	**Ordinary high school**	**Secondary vocational high school**	**Height (cm)**	**Weight (kg)**
A	15–18	12 (30)	17 (30)	1.61 ± 5.16	50 ± 3.7
B	16–18	20 (41)	21 (41)	1.60 ± 5.23	51 ± 4.1
C	16–18	82 (174)	92 (174)	1.62 ± 4.67	50 ± 4.3
D	16–18	46 (103)	57 (103)	1.61 ± 5.55	51 ± 3.9
E	15–18	50 (100)	50 (100)	1.60 ± 6.01	52 ± 4.0

**Figure 2:**
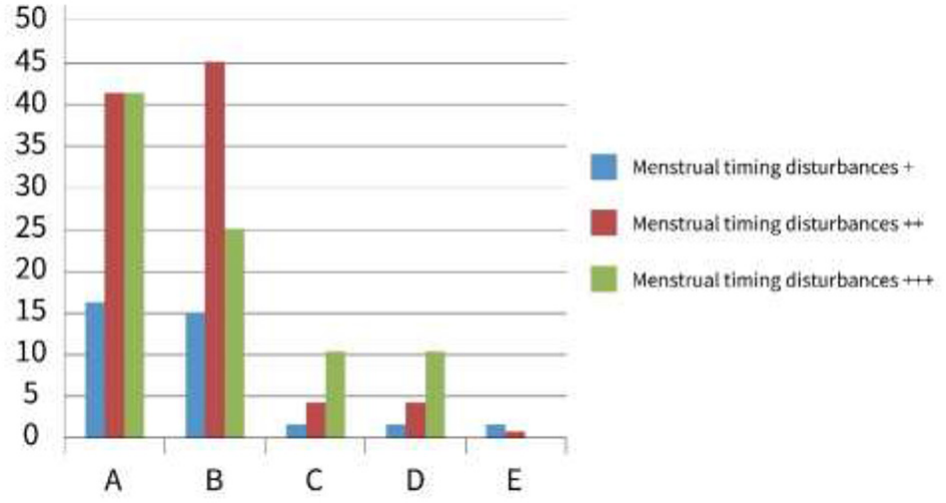
Bar chart comparing menstrual timing disturbances across five categories labeled A to E. Three types of disturbances are shown: blue for +, red for ++, and green for +++. Category B exhibits the highest values, especially for the red and green bars, indicating more severe disturbances. Categories A and B show noticeable differences compared to C, D, and E. Prevalence of circadian rhythm dysregulation among female students in pugao senior high schools. School bullying population mobility developmental stages prevalence of circadian rhythm dysregulation among female students in Pugao senior high schools (%).

Regarding the impact of menstrual irregularities on high school female students during the developmental stage of campus bullying, the results revealed significant differences between group A (general high school) and group B (vocational high school) compared to the control group E (*p* < 0.001). Additionally, there were notable differences between groups C and D (*p* < 0.05) (Table 2, Figure 2). These findings suggest that menstrual irregularities are more pronounced among students who are subjected to campus bullying, particularly those in vocational high schools. In addition, groups C and D, which represent students experiencing different intensities of bullying, showed a similar trend, further supporting the negative impact of bullying on menstrual health.

**Table 2:** Effects of circadian rhythm dysregulation in female high school students during developmental Stages of bullying (incidence/sample size).

**Group**	**Menstrual timing disturbances (+−++)**	**Menstrual timing disturbances (+++)**	**Menstrual volume increases**	**Menstrual volume decreases**	**Abdominal pain and amenorrhea**
A (Ordinary)	7 (12)^#⋇^	5 (12)^#⋇^	4 (12)^#⋇^	5 (12)^#⋇^	2 (12)^#⋇^
A (Secondary)	7 (17)^#⋇^	6 (17)^#⋇^	2 (17)^#⋇^	7 (17)^#⋇^	4 (17)^#⋇^
B (Ordinary)	12 (20)^#⋇^	5 (20)^#⋇^	4 (20)^#⋇^	7 (20)^#⋇^	5 (20)^#⋇^
B (Secondary)	11 (21)^#⋇^	7 (21)^#⋇^	6 (21)^#⋇^	10 (21)^#⋇^	3 (21)^#⋇^
C (Ordinary)	9 (82)^#⋇^	3 (82)	5 (82)^#⋇^	7 (82)^#⋇^	3 (82)
C (Secondary)	11 (92)^#⋇^	5 (92)	7 (92)^#⋇^	5 (92)	2 (92)
D (Ordinary)	3 (46)	5 (46)	1 (46)	3 (46)	2 (46)
D (Secondary)	4 (57)	4 (57)	3 (57)	7 (57)	1 (57)
E (Ordinary)	2 (100)	1 (100)	0 (100)	4 (100)	0 (100)
E (Secondary)	1 (100)	2 (100)	2 (100)	1 (100)	0 (100)

^#⋇^Comparison between group A and group B and control group E: *P* < 0.001, and comparison between group C and control group D: *P* < 0.05. With the different nature of violence in school bullying, the negative impact on menstrual physiology of female high school students in development period is obviously enhanced.

**Figure 3:**
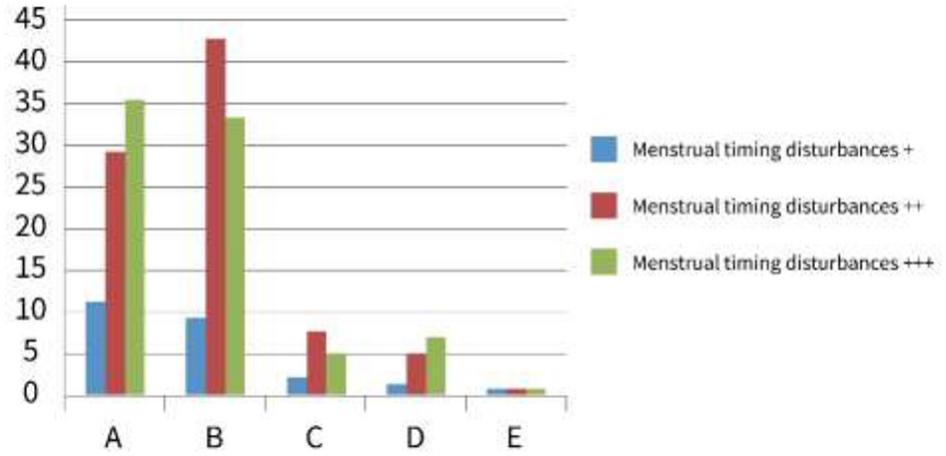
Bar chart showing menstrual timing disturbances across five categories: A, B, C, D, and E. Categories are color-coded for disturbance levels: blue for +, red for ++, and green for +++. Category B shows the highest level of disturbances, especially in red (++), while C, D, and E depict minimal values. Prevalence of circadian dyssynchrony among female vocational high school students. School bullying population mobility developmental stage prevalence of circadian desynchrony among female vocational high school students.

In the case of vocational high schools, significant differences were observed between groups A and B and the control group E (*p* < 0.001), while groups C and D also showed significant differences (*p* < 0.05) (Table 2, Figure 3). This supports the hypothesis that campus bullying exacerbates menstrual disruptions not only in general high schools but also in vocational institutions, where students may face different socioenvironmental pressures.

In terms of visual analog scale (VAS) scores, which measure the psychological impact on high school female students during the developmental stage of campus bullying, there were significant differences between groups A and B and groups C, D, and the control group E (*p* < 0.001). These results underscore the substantial psychological toll bullying takes on students, with higher VAS scores indicating worse psychological outcomes in those subjected to bullying. Furthermore, groups C and D showed significant differences compared to the control group E (*p* < 0.05), suggesting that the psychological effects are particularly prominent among those who experience more intense forms of bullying. As symptoms worsened, the VAS scores significantly increased in the comparison between groups A and B (*p* < 0.01), demonstrating a clear dose–response relationship between the severity of bullying and psychological distress (Table 3). This emphasizes the need for more targeted psychological interventions for students facing prolonged bullying.

**Table 3:** Visual analog scale scores of psychological impact indicators (x ± s, *n* = sample size) among female high school students at developmental stages of bullying.

**Group**		**Mild insomnia**	**Insomnia fatigue**	**Insomnia anxiety**	**Insomnia mood swings**	**Insomnia depression**
A	Ordinary (*n* = 12)	2.1 ± 0.13	4.9 ± 0.35^*#Φ^	8.3 ± 0.67^*#Φ^	14.3 ± 0.76^*#Φ^	17.3 ± 0.77^*#Φ^
	Secondary (*n* = 17)	2.0 ± 0.11	5.1 ± 0.31^*#Φ^	8.7 ± 0.73^*#Φ^	14.7 ± 0.51^*#Φ^	18.2 ± 0.64^*#Φ^
B	Ordinary (*n* = 20)	2.2 ± 0.15	5.5 ± 0.36^*#Φ^	8.7 ± 0.63^*#Φ^	14.1 ± 0.66^*#Φ^	17.9 ± 0.72^*#Φ^
	Secondary (*n* = 21)	2.7 ± 0.35	5.6 ± 0.31^*#Φ^	8.5 ± 0.44^*#Φ^	13.9 ± 0.71^*#Φ^	18.8 ± 0.81^*#Φ^
C	Ordinary (*n* = 82)	1.1 ± 0.3	2.0 ± 0.61	4.1 ± 0.79	5.01 ± 0.96	6.3 ± 0.89
	Secondary (*n* = 92)	1.2 ± 0.51	4.1 ± 0.78	5.0 ± 0.81	5.0 ± 0.90	6.1 ± 0.79
D	Ordinary (*n* = 46)	0.5 ± 0.15	2.1 ± 0.69	4.0 ± 0.66	5.1 ± 0.80	6.8 ± 0.73
	Secondary (*n* = 57)	0.7 ± 0.21	3.2 ± 0.92	4.1 ± 0.81	5.3 ± 0.78	6.9 ± 0.59
E	Ordinary (*n* = 50)	0.5 ± 0.15	1.1 ± 0.29	2.0 ± 0.16	2.1 ± 0.20	2.2 ± 0.23
	Secondary (*n* = 50)	0.5 ± 0.15	1.1 ± 0.29	2.1 ± 0.16	3.3 ± 0.20	3.2 ± 0.23

There is no significant difference between ordinary high school and vocational high school (*P* > 0.05); ^*#^There were significant differences between groups A and B and C, D and control group (*P* < 0.001). There were significant differences between groups C and D and the control group (*P* < 0.05). ^Φ^Compared with group A and group B, the digital visual analogue scale (VAS) score increased significantly with the aggravation of symptoms (*p* < 0.01).

**Figure 4:**
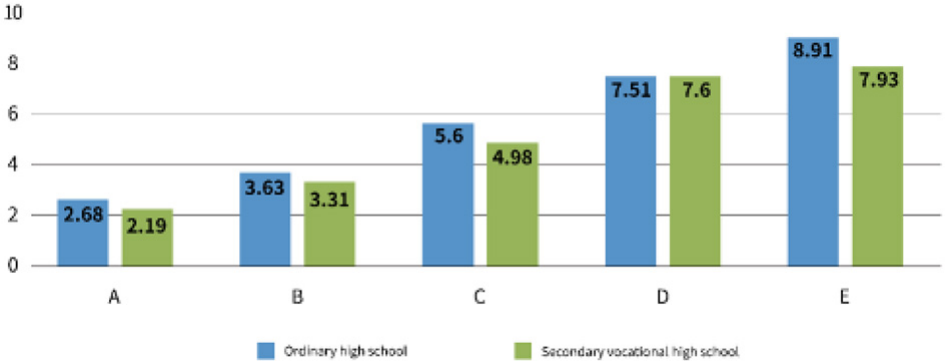
Bar graph comparing scores of ordinary high school and secondary vocational high school across categories A to E. Ordinary high school scores: A - 2.68, B - 3.63, C - 5.6, D - 7.51, E - 8.91. Secondary vocational high school scores: A - 2.19, B - 3.31, C - 4.98, D - 7.6, E - 7.93. Visual analog digital score evaluation of academic performance. Visual analog digital score was evaluated by the evaluation index of academic performance.

Regarding the impact of campus bullying on the learning efficiency of high school female students, VAS scores showed significant differences between groups A and B and the control group E (*p* < 0.001), with significant differences also found between groups C and D compared to the control group E (*p* < 0.05) (Figure 4). These results further suggest that the psychological burden of bullying impacts students' emotional wellbeing and their academic performance, highlighting the multifaceted consequences of bullying on their lives.

## 4 Discussion

This study aimed to examine the physiological and psychological effects of school bullying on adolescent female students, with particular attention to those experiencing population mobility. Our findings largely confirm the hypothesis that bullying, especially violent and multiple-type bullying, is associated with significant menstrual disorders and psychological distress, including depressive symptoms and impaired sleep quality. These results underscore the complex interplay between physiological development and psychosocial stressors during adolescence.

Norway was among the first countries to implement a nationwide survey and anti-bullying campaign, followed closely by the United Kingdom, which has conducted the largest scale of school bullying research with clear evidence of intervention effectiveness. In Norway, bullying incidents have decreased in both primary and junior high schools (Roland, 2000; Stephens, 2011). Similar systematic anti-bullying efforts have been reported in the United States, Spain, Belgium, and Finland, with varying degrees of success in reducing bullying prevalence and mitigating its negative outcomes (Huybregts et al., 2004; Ortega et al., 2004; Salmivalli et al., 2013; Rawlings and Stoddard, 2019). These countries applied multi-level strategies incorporating legislative frameworks, school policies, and community engagement and provided important benchmarks for intervention design. This global effort highlights the critical role of comprehensive anti-bullying programs, which not only address the immediate effects of bullying but also contribute to long-term behavioral and psychological changes in school environments.

The literature indicates that school bullying disproportionately affects female students in the developmental period, with higher prevalence rates compared to their male counterparts. However, this subgroup has historically received limited focused attention as a distinct population in societal and educational discourse (Carlyle and Steinman, 2007; Lapidot-Lefler and Dolev-Cohen, 2015; Li et al., 2020; Pontes et al., 2018). Recent studies suggest that female adolescents could experience bullying differently due to the intersection of gender and developmental stages, which can lead to heightened vulnerability to psychological and physiological disorders (Hysing et al., 2021; Ames and Yon, 2022; Menken et al., 2022; Feng et al., 2024). Bullying in female adolescents significantly undermines self-confidence, self-esteem, and social competence (Brito and Oliveira, 2013; Camodeca et al., 2015; Zhang et al., 2025). These psychosocial impairments are often accompanied by somatic symptoms, including headaches, abdominal pain, insomnia, and nightmares, which are indicative of the embodied nature of bullying-related stress (Figueiredo, 2024). Importantly, menstrual irregularities have emerged as a sensitive physiological marker linked to bullying exposure, highlighting a critical need for integrated approaches to adolescent health that bridge psychological and biological domains (Su et al., 2018; Kanamori et al., 2025; Liao et al., 2025).

Contrary to vague assumptions about “psychological impacts,” recent evidence suggests that bullying triggers a cascade of internalizing disorders, predominantly anxiety and depression, which in turn exacerbate somatic complaints and impair academic functioning (Menken et al., 2022; Xie and Cui, 2022; Zhao et al., 2024). Our findings resonate with a growing body of research emphasizing the bidirectional and resonant effects between mental health disturbances and physiological dysregulation in bullied youths. The cyclical nature of this deterioration underscores the urgency of early detection and targeted intervention.

From a developmental perspective, adolescence is a crucial period for the maturation of female reproductive physiology, with menarche typically occurring between ages 12 to 14, followed by hormonal stabilization during late adolescence. Stressors such as bullying during this period can disrupt hypothalamic–pituitary–ovarian axis functioning, leading to menstrual cycle irregularities and associated psychosocial distress (Su et al., 2018). This disruption may have downstream effects on social gender identity formation and psychological development, further complicating the adolescent experience.

Our study identified alarming rates of severe menstrual disorders among female students experiencing violent and multiple types of bullying, with incidences of delayed or early menstruation reaching up to 58.3% and 41.6%, respectively. Additional symptoms, such as increased or decreased menstrual volume and severe abdominal pain, were prevalent, corroborating findings from similar populations internationally (Liao et al., 2025). These physiological markers align with psychological distress indices, forming a measurable and clinically relevant profile of the impact of bullying.

Furthermore, the digital visual analog scale (VAS) employed demonstrated sensitivity in quantifying academic performance disruptions related to bullying exposure. Students in bullying groups exhibited significantly lower academic stability scores, with implications for educational attainment and social mobility (Menken et al., 2022; Xie and Cui, 2022). This finding underscores the potential of using digital tools for real-time monitoring and intervention in school settings.

The digital VAS developed in this study effectively quantifies the severity of bullying-related impairments in both academic performance and physiological status, thereby providing a sensitive tool for early detection and monitoring. The scale's ability to capture gradations in psychological and somatic symptoms aligns with clinical assessment standards such as the Hamilton Depression Rating Scale (Faravelli et al., 1986; May and Pridmore, 2020), yet offers the advantage of applicability in school settings for timely identification.

Parents, educators, and healthcare providers must be vigilant in recognizing early somatic complaints linked to bullying, which often precede overt psychological crises. Psychogenic symptoms, such as headaches, stomachaches, and menstrual irregularities, frequently prompt clinical visits. However, without the awareness of the underlying bullying-related causes, these symptoms risk misclassification and undertreatment. The bidirectional deterioration of mental and physical health in bullied adolescents necessitates integrated biopsychosocial interventions to interrupt negative spirals and improve outcomes.

Finally, failing to detect and intervene during the early stages of bullying-induced distress contributes to heightened rates of anxiety, depression, academic decline, severe menstrual disorders, and, in extreme cases, self-harm and suicidality. Our study contributes to this critical evidence base by demonstrating measurable physiological correlates of bullying alongside psychological impairments, thereby advocating for comprehensive school-based health programs and community engagement to mitigate these adverse trajectories.

## 5 Conclusion

This study provides robust evidence that school bullying, particularly violent and multiple-type bullying, significantly compromises both physiological and psychological health in female adolescents experiencing population mobility. Menstrual irregularities and psychological distress, including depressive symptoms and impaired sleep quality, are prevalent among bullied students, highlighting the intertwined nature of biological and mental health challenges during adolescence. The validated digital visual analog scale (VAS) proved effective in quantifying these multi-dimensional impacts and could serve as a practical tool for early detection and intervention. These findings underscore the urgent need for targeted prevention programs and integrated health services tailored to vulnerable migrant adolescent populations to promote equitable developmental outcomes.

## 6 Implications

The results have important implications for policy, education, and clinical practice. Schools should implement comprehensive anti-bullying strategies that address the unique vulnerabilities of migrant female students, integrating psychosocial support with reproductive health monitoring. Educators and healthcare providers must collaborate to develop screening protocols using tools such as the digital VAS for early identification of at-risk individuals. Moreover, policymakers should prioritize resource allocation to migrant youth health programs, recognizing the compounded risks associated with migration and bullying. Holistic interventions that encompass physical, mental, and social dimensions are critical for fostering resilience and academic success among this marginalized group.

## 7 Limitations

Several limitations warrant consideration. First, the observational design precludes causal inference, though longitudinal data strengthen temporal associations. Second, bullying exposure was assessed via proxy report, which could introduce reporting bias despite efforts to preserve confidentiality. Third, the sample, although robust, represents only certain regions in China, limiting generalizability to broader populations. Fourth, potential confounders such as the family's socioeconomic status and school climate factors were not fully controlled. Finally, physiological measurements were limited to menstrual and electrophysiological indicators, excluding other relevant biomarkers.

## 8 Future directions

Future studies should employ mixed-method approaches to deepen the understanding of the mechanisms that link bullying and adolescent health in migrant populations. Expanding geographic scope and sample diversity will enhance generalizability. Incorporating objective biomarkers, such as hormonal assays and stress-related physiological parameters, could elucidate underlying biological pathways. Intervention studies testing the effectiveness of integrated psychosocial and reproductive health programs are urgently needed. Additionally, exploring the role of family, peer, and community support systems in buffering the impact of bullying will help in planning multi-level prevention strategies. Developing culturally sensitive and gender-specific tools for assessment and intervention remains a critical priority.

## Data Availability

The data analyzed in this study is subject to the following licenses/restrictions: Given its sensitive nature, access to the dataset is restricted to authorized personnel involved in the research. External parties or the public do not have access to the dataset. All handling of the data adheres to strict data protection policies and ethical guidelines. Requests to access these datasets should be directed to yifeili@ruc.edu.cn.
