# Correction: Negative correlation between school bullying and multi-dimensional health in adolescent female migrants: a digital visual analog scale study

**DOI:** 10.3389/fpsyg.2025.1725342

**Published:** 2025-11-07

**Authors:** Yueping Song, Yifei Li, Kai Zhou, Jia Tang, Xuemeng Chen, Gaowang Liu

**Affiliations:** 1 Research Center for Social and Population Development, Renmin University of China, Beijing, China; 2 School of Population and Health, Renmin University of China, Beijing, China; 3 Big Data and Responsible Artificial Intelligence for National Governance, Renmin University of China, Beijing, China; 4 School of International Trade and Economics, Central University of Finance and Economics, Beijing, China; 5 Policy Research Center for Environment and Economy, Ministry of Ecology and Environment of the People's Republic of China, Beijing, China; 6 Department of Anesthesiology, Deyang People's Hospital, Deyang, Sichuan, China; 7 Department of Anesthesiology, Nanfang Hospital, Southern Medical University, Guangzhou, Guangdong, China; 8 Key Laboratory of Precision Anesthesia & Perioperative Organ Protection, Guangzhou, Guangdong, China

**Keywords:** school bullying, female high school students, physiological indicators, psychological distress, digital visual analog scale (VAS), China

Author Yifei Li was erroneously assigned to affiliation 2. School of Population and Health, Renmin University of China, Beijing, China. This affiliation has now been removed for author Yifei Li.

The original version of this article has been updated.

